# Risk Factors for the Development of Pneumonia in Stroke Patients: A Systematic Review and Meta-Analysis

**DOI:** 10.7759/cureus.57077

**Published:** 2024-03-27

**Authors:** Mansoor Ahmad, Zeeshan Ayaz, Tanya Sinha, Thin M Soe, Nimish Tutwala, Alahed A Alrahahleh, Divine Besong Arrey Agbor, Neelum Ali

**Affiliations:** 1 Medicine, Rehman Medical Institute, Peshawar, PAK; 2 Medical Education, Tribhuvan University, Kirtipur, NPL; 3 Medicine, University of Medicine 1, Yangon, Yangon, MMR; 4 Obstetrics and Gynaecology, Topiwala National Medical College & B. Y. L. Nair Charitable Hospital, Mumbai, IND; 5 Faculty of Medicine, Yarmouk University, Irbid, JOR; 6 Clinical Research and Internal Medicine, California Institute of Behavioral Neurosciences & Psychology, Fairfield, USA; 7 Internal Medicine, Richmond University Medical Center, Staten Island, USA; 8 Internal Medicine, University of Health Sciences, Lahore, PAK

**Keywords:** systematic review, hospitalization, pneumonia, stroke, predictors

## Abstract

Pneumonia is one of the most prevalent medical complications post-stroke. It can have negative impacts on the prognosis of stroke patients. This study aimed to determine the predictors of pneumonia in stroke patients. The authors devised, reviewed, and enhanced the search strategy in accordance with the Preferred Reporting Items for Systematic Reviews and Meta-Analyses (PRISMA) guidelines. Studies were gathered from various electronic databases, including Medline, CINAHL, Cochrane, Embase, and Web of Science, from January 1st, 2011, to February 25th, 2024. The review encompassed studies involving patients aged 18 years and older who were hospitalized for acute stroke care. Inclusion criteria required patients to have received a clinical diagnosis of stroke, confirmed via medical imaging (CT or MRI), hospital primary diagnosis International Classification of Diseases 10th Revision discharge codes, or pathology reporting. A total of 35 studies met the criteria and were included in our pooled analysis. Among them, 23 adopted a retrospective design, while the remaining 12 were prospective. The pooled incidence of pneumonia among patients with stroke was found to be 14% (95% confidence interval = 13%-15%). The pooled analysis reported that advancing age, male gender, a history of chronic obstructive pulmonary disease (COPD), the presence of a nasogastric tube, atrial fibrillation, mechanical ventilation, stroke severity, dysphagia, and a history of diabetes were identified as significant risk factors for pneumonia development among stroke patients. Our results underscore the importance of proactive identification and management of these factors to mitigate the risk of pneumonia in stroke patients.

## Introduction and background

Stroke presents a significant global health challenge, impacting millions and resulting in substantial morbidity and mortality [1]. According to the World Health Organization, stroke ranks as the second leading cause of death worldwide and the primary cause of disability in high-income nations [2]. Post-stroke pneumonia emerges as a common and severe complication following a stroke [3]. It refers to the occurrence of pneumonia in individuals who have recently experienced a stroke, often attributable to compromised immunity and a weakened cough reflex in stroke patients [4]. Pneumonia is one of the most prevalent medical complications post-stroke, with reported incidence rates ranging from 5% to 26% [5]. Post-stroke pneumonia not only extends hospital stays and escalates healthcare costs but also significantly impacts patient outcomes [6]. It correlates with heightened mortality rates, increased risk of hospital readmission, prolonged length of stay, and diminished functional outcomes. Moreover, post-stroke pneumonia can impede the rehabilitation process and hinder the recovery journey of stroke patients [7].

Research indicates that the occurrence of pneumonia during the initial phase of a stroke is notably elevated in older patients with underlying health conditions such as heart failure, chronic obstructive pulmonary disease (COPD), and widespread atherosclerosis. Furthermore, the prevalence of dysphagia, frequently observed following a stroke, heightens the likelihood of aspiration and consequent pneumonia [8]. While the precise mechanisms by which post-stroke pneumonia impacts patient outcomes remain incompletely elucidated, potential contributing factors encompass fever, hypoxia, hypotension, as well as the activation of leukocytes and platelets [9].

Differences in study methodologies pose challenges in comparing findings and conducting comprehensive meta-analyses on this subject. To our knowledge, only one systematic review, inclusive of a meta-analysis of observational studies exploring the risk factors for pneumonia in stroke patients, has been reported thus far [10]. However, subsequent to this review, numerous new studies, both prospective and retrospective observational studies, have emerged. Hence, there is a pressing need for an updated systematic review and meta-analysis to identify the factors linked with the onset of pneumonia in stroke patients.

## Review

Methodology

The authors devised, reviewed, and enhanced the search strategy in accordance with the Preferred Reporting Items for Systematic Reviews and Meta-analyses (PRISMA) guidelines. Studies were gathered from various electronic databases, including Medline/PubMed, CINAHL, Cochrane, EMBASE, and Web of Science, from January 1st, 2011, to February 25th, 2024. Search terms were selected based on common terms and synonyms for stroke, pneumonia, and associated risk factors. These terms were adjusted across databases to maintain sensitivity and ensure an appropriate search output. Additionally, reference lists of identified manuscripts and review articles underwent scrutiny. Two authors independently conducted the search, with any discrepancies resolved through discussion.

Study Selection

The review encompassed studies involving patients aged 18 years and older who were hospitalized for acute stroke care. Inclusion criteria required patients to have received a clinical diagnosis of stroke, confirmed via medical imaging (CT or MRI), hospital primary diagnosis International Classification of Diseases 10th Revision discharge codes, or pathology reporting. Studies exclusively focusing on patients with pre-existing medical conditions unrelated to stroke, which could predispose them to chest infections (e.g., cystic fibrosis, bronchiectasis, or lung cancer), were excluded. Additionally, studies including patients with a prior diagnosis of pneumonia before admission were not considered for this meta-analysis. Observational studies, including both prospective and retrospective cohort studies, evaluating at least one risk factor for pneumonia subsequent to stroke, were eligible for inclusion. We excluded studies that included transient ischemic attack patients. However, cohort studies primarily investigating the association between a specific intervention and outcomes, rather than risk factors, were excluded, as were laboratory-based studies. Studies exploring biochemical risk factors, particularly blood markers, were also omitted from consideration.

Full-text articles published in the English language within peer-reviewed journals were considered eligible. The combined output from all databases was aggregated, and duplicate entries were removed. Screening for eligibility was conducted based on the title and abstract. This screening process was independently performed by two authors, with any uncertainty regarding eligibility resolved through consensus with co-authors. Studies that could not be conclusively assessed based solely on their title or abstract underwent retrieval of their full text to ascertain eligibility.

Data Extraction and Quality Assessment

Data extraction was performed using a standardized data extraction sheet developed using Microsoft Excel. Data were extracted by two authors independently. Data extracted from eligible studies were author name, publication year, study region, study design, total sample size, number of patients with stroke who developed pneumonia, and factors associated with pneumonia. Any disagreement in the process of data extraction was resolved through discussion. Quality assessment was performed using the Newcastle-Ottawa Scale. This scale systematically assesses various aspects of study design, including participant selection, comparability of study groups, and ascertainment of exposure or outcome, to provide a comprehensive evaluation of the study’s reliability and validity.

Statistical Analysis

Predictors for a particular outcome were consolidated if data from at least three articles were available. Analysis was conducted using a random-effects model. The overall combined odds ratio (OR) with a 95% confidence interval (CI) and corresponding p-value were computed for categorical variables and mean difference (MD) with 95% CI were computed for continuous variables. Predictors with p-values <0.05 were deemed statistically significant. Heterogeneity among studies was assessed using I^2^, with a value greater than 30% indicating moderate to high heterogeneity. Data analysis was executed using RevMan Version 5.4.1 (The Cochrane Collaboration, London, United Kingdom). We used STATA version 17.0 (StataCorp., College Station, TX, USA) to determine the pooled incidence of post-stroke pneumonia using the MetaProp command.

Results

**Figure 1:**
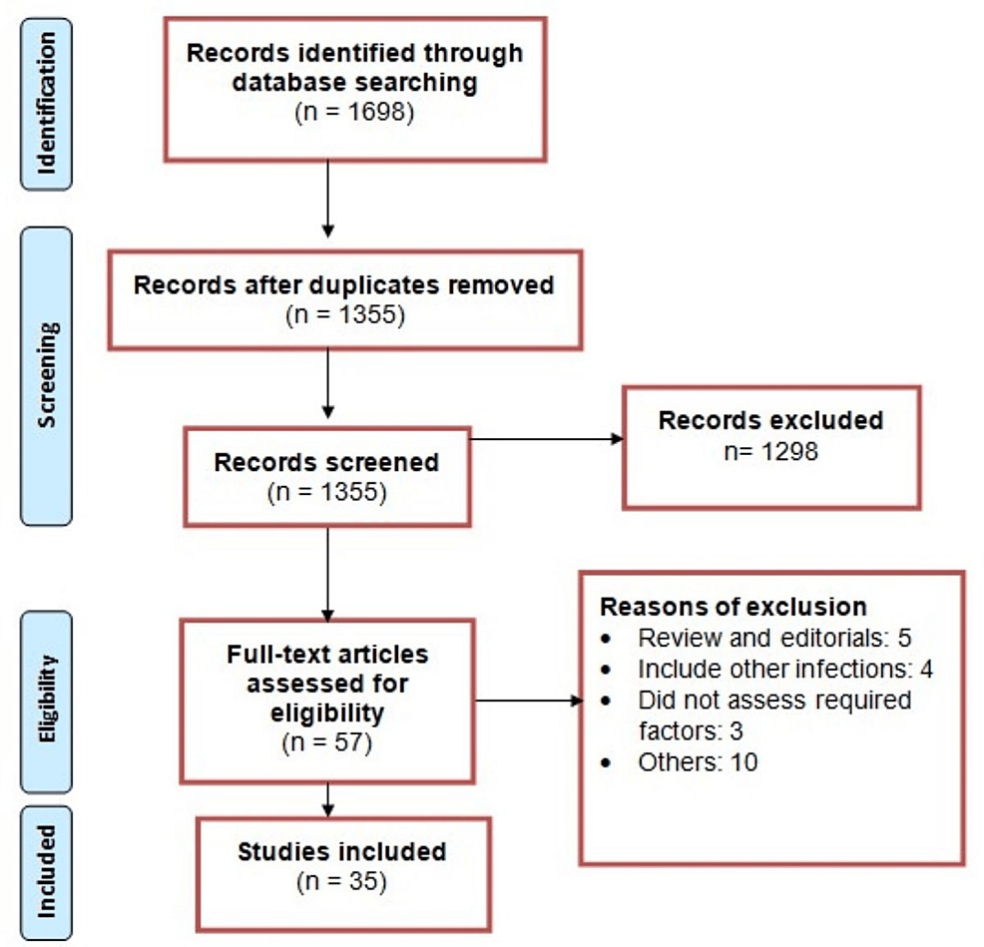
Preferred Reporting Items for Systematic Reviews and Meta-Analyses flowchart of study selection.

Figure 1 shows the process of study selection. We conducted a systematic search of online databases, identifying a total of 1,698 records. Following the elimination of duplicates, we initially screened 1,355 articles based on their abstracts and titles. Subsequently, 57 studies underwent a comprehensive evaluation of their full texts, wherein they were rigorously assessed against predefined inclusion and exclusion criteria. Ultimately, 35 studies met the criteria and were included in our pooled analysis, aimed at elucidating the factors contributing to pneumonia development in stroke patients. Table 1 provides an overview of the characteristics of these included studies. Among them, 23 adopted a retrospective design, while the remaining 12 were prospective. Notably, a significant proportion of the studies were conducted in China (n = 11), with the United States contributing the second-highest number of studies (n = 5). Additionally, research was conducted across various regions, encompassing Germany, Brazil, Taiwan, Thailand, Korea, Nigeria, Ethiopia, Chile, Canada, Austria, and Poland. The pooled incidence of pneumonia among patients with stroke was found to be 14% (95% CI = 13% to 15%). Across individual studies, the incidence of pneumonia ranged from 8% to 38%. To further delineate these findings, a subgroup analysis was conducted based on the study design. The pooled analysis of retrospective studies revealed a pneumonia incidence of 12% (95% CI = 11% to 14%), while prospective studies yielded a pooled incidence of 20% (95% CI = 14% to 25%) among stroke patients. Table 2 presents the quality assessment of the included studies.

**Table 1:** Characteristics of included studies.

Author(s)	Year	Region	Study design	Total population	Number of participants who developed pneumonia
Akimoto et al. [11]	2023	Japan	Retrospective	281	24
Almeida et al. [12]	2015	Brazil	Retrospective	159	51
Alsumrain et al. [13]	2012	United States	Prospective	280	39
Assefa et al. [14]	2022	Ethiopia	Prospective	325	116
Barlas et al. [15]	2019	Thailand	Retrospective	610,668	58,586
Bruening and Al-Khaled [16]	2015	Germany	Prospective	538	122
Chen et al. [17]	2012	Taiwan	Retrospective	341	23
Colbert et al. [18]	2016	United States	Retrospective	91,643	1,225
Divani et al. [19]	2015	United States	Retrospective	591	116
Finlayson et al. [20]	2011	Canada	Retrospective	8,251	587
Hoffmann et al. [21]	2012	Germany	Retrospective	15,335	1,104
Hoffmeister et al. [22]	2013	Chile	Retrospective	677	167
Huang et al. [23]	2019	China	Prospective	643	70
Ji et al. [24]	2013	China	Retrospective	8,820	1,007
Jitpratoom et al. [25]	2024	Thailand	Retrospective	342	54
Lee et al. [26]	2023	Korea	Retrospective	367	54
Li et al. [27]	2014	China	Prospective	1,142	215
Li et al. [28]	2022	China	Prospective	2,366	459
Liang et al. [29]	2022	China	Retrospective	790,811	64,398
Liao et al. [30]	2015	China	Retrospective	221,254	14,185
Lidetu et al. [31]	2023	Ethiopia	Retrospective	568	131
Maeshima et al. [32]	2014	Japan	Prospective	254	14
Masrur et al. [33]	2013	United States	Prospective	314,007	17,906
Matz et al. [34]	2016	Austria	Retrospective	59,558	3,111
Patel et al. [35]	2020	United States	Retrospective	4,224,924	149,169
Sadiq et al. [36]	2023	Nigeria	Retrospective	591	102
Schaller-Paule et al. [37]	2022	Germany	Retrospective	4,281	832
Sui et al. [38]	2011	China	Retrospective	1,435	545
Szylińska et al. [39]	2022	Poland	Prospective	1,001	227
Tashima et al. [40]	2023	Japan	Retrospective	340	22
Yamamoto et al. [41]	2014	China	Retrospective	133	9
Yu et al. [42]	2016	Taiwan	Retrospective	934	100
Yuan et al. [43]	2021	China	Prospective	451	98
Zhang et al. [44]	2012	China	Prospective	106	32
Zhang et al. [45]	2023	China	Prospective	248	83

**Table 2:** Quality assessment of included studies using the Newcastle-Ottawa scale.

Author(s)	Selection	Comparison of groups	Assessment of outcome and exposure	Overall
Akimoto et al. [11]	3	2	3	Good
Almeida et al. [12]	3	2	3	Good
Alsumrain et al. [13]	3	2	2	Fair
Assefa et al. [14]	3	1	3	Fair
Barlas et al. [15]	3	2	2	Fair
Bruening and Al-Khaled [16]	2	2	3	Fair
Chen et al. [17]	2	1	3	Fair
Colbert et al. [18]	4	2	3	Good
Divani et al. [19]	3	1	3	Fair
Finlayson et al. [20]	3	1	3	Fair
Hoffmann et al. [21]	3	2	2	Fair
Hoffmeister et al. [22]	3	2	3	Good
Huang et al. [23]	3	2	3	Good
Ji et al. [24]	2	2	2	Fair
Jitpratoom et al. [25]	3	2	3	Good
Lee et al. [26]	3	1	3	Fair
Li et al. [27]	4	2	3	Good
Li et al. [28]	3	2	3	Good
Liang et al. [29]	2	2	3	Fair
Liao et al. [30]	4	1	3	Good
Lidetu et al. [31]	3	2	3	Good
Maeshima et al. [32]	4	1	3	Good
Masrur et al. [33]	2	2	3	Fair
Matz et al. [34]	2	2	3	Fair
Patel et al. [35]	4	2	2	Good
Sadiq et al. [36]	3	2	3	Good
Schaller-Paule et al. [37]	4	2	3	Good
Sui et al. [38]	3	2	3	Good
Szylińska et al. [39]	3	2	2	Fair
Tashima et al. [40]	4	1	3	Good
Yamamoto et al. [41]	4	2	2	Good
Yu et al. [42]	4	2	3	Good
Yuan et al. [43]	2	1	3	Fair
Zhang et al. [44]	3	2	3	Good
Zhang et al. [45]	4	2	3	Good

Predictors of Pneumonia in Stroke Patients

Table 3 delineates the outcomes pertaining to factors correlated with the onset of pneumonia among stroke patients admitted to hospital settings. As illustrated in Table 3, there was a notable disparity in mean age between patients who developed pneumonia and those who did not, with the former exhibiting significantly higher age averages. Regarding gender distribution, the likelihood of pneumonia occurrence was markedly higher among male patients in comparison to their female counterparts.

**Table 3:** Predictors of pneumonia in stroke patients. ^: Presented as mean difference (95% CI). OR = odds ratio; CI = confidence interval; COPD = chronic obstructive pulmonary disease; BMI = body mass index; NIHSS = National Institutes of Health Stroke Scale

Variable	OR (95% CI)	P-value	I^2^
Age^	5.24 (4.24 to 6.18)	0.001	100%
Gender (male)	1.09 (1.04 to 1.15)	0.001	91%
BMI^	-0.02 (-0.56 to 0.52)	0.94	36%
Smoking	1.01 (0.87 to 1.16)	0.91	94%
Hypertension	1.14 (0.96 to 1.34)	0.14	99%
Diabetes	1.19 (1.10 to 1.29)	0.001	95%
COPD	2 (1.68 to 2.37)	0.001	91%
Atrial fibrillation	2.75 (2.16 to 3.51)	0.001	95%
Dysphagia	10.43 (4.33 to 25.11)	0.001	98%
History of stroke	1.20 (1.13 to 1.28)	0.001	80%
Stroke type (ischemic)	0.41 (0.16 to 1.03)	0.06	92%
Ventilator	8.99 (4.80 to 16.81)	0.001	59%
Nasogastric tube	12.48 (6.37 to 24.43)	0.001	96%
NIHSS score^	6.19 (4.80 to 7.57)	0.001	91%

Aggregate analysis revealed a noteworthy elevation in the risk of pneumonia among patients with diabetes, as opposed to their non-diabetic counterparts (OR = 1.19, 95% CI = 1.10 to 1.29). Similarly, patients diagnosed with atrial fibrillation demonstrated a substantially increased susceptibility to pneumonia (OR = 2.75, 95% CI = 2.16 to 3.51) relative to those without the condition. Additionally, COPD emerged as a significant contributing factor to pneumonia development in stroke patients (OR = 2, 95% CI = 1.68 to 2.37).

Dysphagia also emerged as a significant predictor of pneumonia incidence in stroke patients (OR = 10.43, 95% CI = 4.33 to 25.11). Furthermore, patients with ischemic stroke displayed a trend toward lower pneumonia risk, although this discrepancy did not reach statistical significance (OR = 0.41, 95% CI = 0.16 to 1.03). Notably, patients with nasogastric tube insertion faced a substantially heightened risk of pneumonia compared to those without such intervention (OR = 12.48, 95% CI = 6.37 to 24.43).

Moreover, higher National Institutes of Health Stroke Scale scores were associated with an augmented risk of pneumonia development. Conversely, factors such as smoking history, hypertension, and body mass index did not exhibit statistically significant differences between patients who developed pneumonia and those who did not.

Discussion

This meta-analysis aimed to assess the prevalence of post-stroke pneumonia and identify associated factors. The findings revealed a pooled incidence rate of 14% for post-stroke pneumonia. Notably, advancing age, male gender, a history of COPD, the presence of a nasogastric tube, atrial fibrillation, mechanical ventilation, stroke severity, dysphagia, and a history of diabetes were identified as significant risk factors for pneumonia development among stroke patients. These results are consistent with previous research, as highlighted by Wästfelt et al.’s meta-analysis [10], which also reported similar associations between these factors and pneumonia development in stroke patients.

Of these factors, mechanical ventilation emerged as the strongest predictor of pneumonia onset. All included studies demonstrated an elevated risk of pneumonia among mechanically ventilated patients. This intervention is often necessary for individuals with severe strokes, either due to pulmonary complications or neurological deterioration, albeit associated with high mortality rates [46]. Prolonged supine positioning, intubation, and mechanical ventilation can compromise normal mucociliary clearance, fostering bacterial colonization. Such impaired clearance mechanisms heighten susceptibility to chest infections, notably pneumonia [47]. Implementing protective measures, such as respiratory physiotherapy during mechanical ventilation, early mobilization once deemed safe, and vigilant monitoring upon return to the ward, may mitigate the risk of chest infections in this vulnerable population [48].

Pre-existing respiratory conditions, such as COPD, can elevate the susceptibility to pneumonia. COPD is characterized by reduced alveolar elasticity, thereby compromising mucociliary clearance [49]. Moreover, a significant proportion of COPD patients are smokers, further compromising their immune defenses. Consistent with this, our meta-analysis indicated a heightened risk of pneumonia among smokers compared to non-smokers. These combined factors create conducive environments for pathogen colonization within the respiratory tract, thereby increasing the likelihood of chest infections, which, when coupled with the additional risk posed by stroke, accentuates the overall vulnerability [49].

Furthermore, our meta-analysis unveiled a greater predisposition for post-stroke pneumonia among males. This gender disparity may reflect genuine differences in incidence rates between males and females [50], possibly attributed to the higher prevalence of current and former smoking habits among males in these age cohorts [51].

Diabetes demonstrated an association with the onset of pneumonia following a stroke. Individuals with diabetes face an elevated risk of underlying cardiovascular ailments and complications such as peripheral neuropathy and peripheral artery disease. These conditions may hinder a patient’s ability to tolerate mobilization post-stroke and may detrimentally impact their overall health and potential for recovery [52]. Mobilization plays a pivotal role in optimizing oxygen delivery and facilitating airway clearance to mitigate complications such as chest infections [53]. Thus, endeavors to mitigate hospital-acquired complications in patients with diabetes are crucial.

Similarly, atrial fibrillation exhibited a modest correlation with the development of chest infections, a finding consistent across all studies. Several factors contribute to this heightened risk. First, atrial fibrillation is linked with increased stroke severity, such as total anterior circulation syndrome, which may heighten the risk of aspiration, consequently leading to pneumonia [39]. Second, advanced age is a common risk factor for both atrial fibrillation and pneumonia. Older patients may contend with baseline cognitive impairment and immunodepression, rendering them more susceptible to infections such as pneumonia. Additionally, atrial fibrillation frequently coexists with other comorbidities such as diabetes and COPD, both identified as risk factors for pneumonia in this meta-analysis [25].

Dysphagia exhibited a modest correlation with the development of chest infections, a consistent finding across all studies. Dysphagia, characterized by difficulty in drinking, chewing, swallowing, or safeguarding the airway, commonly manifests following a stroke [54]. Swallowing difficulties may precipitate aspiration, potentially causing irritation or damage to the lungs. Up to one-third of stroke patients who aspirate may subsequently develop pneumonia [55].

The utilization of a nasogastric tube in stroke patients has been linked to an elevated risk of pneumonia development. This association may stem from various factors. First, the presence of a nasogastric tube can impede proper swallowing, heightening the likelihood of aspiration [55]. This assertion is corroborated by studies indicating a heightened incidence of pneumonia among stroke patients receiving nasogastric tube feedings. Second, nasogastric tube insertion can induce respiratory tract trauma, disrupting the airway’s natural defense mechanisms and augmenting susceptibility to respiratory infections such as pneumonia [56]. Moreover, prolonged nasogastric tube usage can precipitate complications such as nasal wing lesions, chronic sinusitis, and gastroesophageal reflux, further amplifying the pneumonia risk [57].

Early recognition of individuals with pre-existing respiratory conditions, cardiac ailments including atrial fibrillation, or diabetes could potentially mitigate the risk of chest infections post-stroke by ensuring prompt and effective management of these pre-existing or concomitant medical conditions during the acute phase. The findings of this review underscore the imperative for heightened vigilance upon admission regarding comorbidities and intensified monitoring of respiratory status, particularly among patients undergoing mechanical ventilation or those with pre-existing respiratory or cardiac conditions, including atrial fibrillation. Furthermore, there is a pressing need for additional high-quality research focusing on modifiable risk factors such as current smoking and pre-existing cardiac conditions to generate homogeneous data, facilitating further meta-analyses. With early identification of individuals susceptible to chest infections, further exploration into prophylactic interventions for these patients is warranted.

Research limitations

The meta-analysis conducted on post-stroke pneumonia revealed considerable levels of heterogeneity among the included studies, indicating potential disparities that render them not entirely comparable. This variability may stem, in part, from the inclusion of different independent variables in the regression models across studies. Additionally, the majority of articles focused on pneumonia or lung infections during hospitalization, whereas the remaining articles examined pneumonia within a longer time frame (ranging from 4 days to 30 days post-stroke). Furthermore, the studies were incorporated irrespective of their specific definitions of pneumonia, potentially contributing to heterogeneity stemming from differences in how pneumonia was characterized.

Given the nature of this review, which exclusively incorporated observational studies, it is important to note that while this study design is well-suited for identifying risk factors, it does not permit causal inferences. Consequently, it is not feasible to definitively assert that the identified risk factors directly cause pneumonia; rather, it can only be concluded that associations between the identified risk factors and pneumonia exist.

## Conclusions

The incidence of pneumonia in stroke patients was 14%. Among the findings, advanced age, male gender, comorbidities such as diabetes and COPD, dysphagia, and interventions such as mechanical ventilation and nasogastric tube insertion emerged as substantial risk factors. Our results underscore the importance of proactive identification and management of these factors to mitigate the risk of pneumonia in stroke patients. However, the presence of heterogeneity among studies and the observational nature of the research warrant cautious interpretation of the findings and call for further high-quality investigations to strengthen evidence-based interventions.
